# Arabic version of the SF-Qualiveen: cross-cultural adaptation, translation, and validation of urinary disorder‐specific instruments in patients with multiple sclerosis

**DOI:** 10.1186/s12894-024-01429-4

**Published:** 2024-02-12

**Authors:** Younes A. Khadour, Meng Zheng, Fater A. Khadour

**Affiliations:** 1 Department of Physical Therapy, Cairo University, Cairo, 11835 Egypt; 2 Department of Rehabilitation, Faculty of Medicine, Al Baath University, Homs, Syria; 3 Department of Orthopaedic Surgery, Tongji Hospital, Tongji Medical College, Huazhong University of Science and Technology, Wuhan, 430030 China; 4 Department of Physical Therapy, Health Science Faculty, Al-Baath University, Homs, Syria; 5 Department of Rehabilitation, Tongji Hospital, Tongji Medical College, Huazhong University of Science and Technology, 1095#, Jie-Fang Avenue, Qiaokou District, Wuhan, 430030 Hubei China

**Keywords:** The SF-Qualiveen Questionnaire, Quality of life, Neurogenic lower urinary tract dysfunction, Reliability, Validity

## Abstract

**Background:**

The Short Form Qualiveen (SF-Qualiveen) questionnaire assesses the effect of bladder and urinary symptoms on patients’ quality of life (QoL) with urological impairment caused by neurological diseases. There is no validated SF-Qualiveen questionnaire in Arabic, so this study aims to provide a translated and validated version of the SF-Qualiveen questionnaire among Arabic-speaking patients with multiple sclerosis (MS).

**Methods:**

The English version of the SF-Qualiveen was translated into Arabic using an algorithm for linguistic and cultural adaptation. MS patients completed the SF-Qualiveen, and the Neurogenic Bladder Symptom Score(NBSS) questionnaire. Psychometric features such as content and construct validity, test–retest reliability, and internal consistency were analyzed. Construct validity was evaluated by contrasting the SF-Qualiveen with the NBSS questionnaire. Internal consistency was measured using Cronbach’s alpha, whereas the intraclass correlation coefficient (ICC) was employed to assess the test-retest reliability.

**Results:**

One hundred and two patients with MS were included in this study. The internal consistency of the total SF-Qualiveen, and the domains “Bother with limitations,” “Fear,” “Feeling,” and “Frequency of limitations” showed good internal consistency (Cronbach’s alpha of > 0.7). ICC was 0.91 for the total score 0.85 for the Bother with limitations, 0.81 for Fears, 0.86 for Feeling, and 0.81 for Frequency of limitations. The correlation analysis revealed a positive association between the total scores on the NBSS and the domains of the SF-Qualiveen, comprising bother with limitations (*r* = 0.473, *p* = 0.027), fears (*r* = 0.611, *p* = 0.031), feelings (*r* = 0.572, *p* = 0.04), and frequency of limitations (*r* = 0.514, *p* = 0.013).

**Conclusions:**

The findings of this validation study revealed that the SF-Qualiveen is a reliable and valid instrument appropriate for Arabic-speaking patients with MS in both research and clinical practice.

## Introduction

Urological dysfunction is common in patients with multiple sclerosis (MS), with prevalence reported as high as 32–97% [1]. This variance is at least partly related to the stage of the progression of the disease. While overactive bladder (OAB) and bladder sphincter dyssynergia (BSD) have been identified as early stage manifestations of MS. OAB refers to a syndrome characterized by urinary urgency, frequency, and sometimes urinary incontinence. BSD, on the other hand, is a condition in which there is a lack of coordination between the contraction of the bladder and relaxation of the urinary sphincter during voiding. These bladder dysfunctions often develop in the early stages of MS, as the disease progresses and affects the neural pathways involved in bladder control. Several studies have demonstrated the prevalence and impact of OAB and BSD in MS patients. A study by de Sèze et al. investigated the prevalence of lower urinary tract symptoms in MS patients and found that OAB was present in 46% of the participants [1]. Another study by C Solaro et al. reported a high prevalence of BSD in MS patients, with approximately 30% of the participants showing signs of sphincter dyssynergia [2]. Lower urinary tract dysfunction symptoms (LUTS) are associated with an important worsening of the quality of life (QoL) in patients with MS [3]. As variations were observed between the perceptions of patients and doctors regarding the effect of neurological conditions like MS, direct assessments of the patient’s suffering and experiences are required to learn about their impression of the disease’s effect on the QoL [4]. The European Association of Urology (EAU) recommendations on neuro-urology emphasize the importance of quality of life in treating patients’ neuro-urological [5].

Urologists frequently utilize bladder diaries and several self-administered questionnaires to get a more precise and comprehensive record of symptoms of lower urinary tract dysfunction patients. The EAU advises using a validated instrument to assess the quality of life of LUTS patients [5]. More than thirty validated questionnaires are designed to assess the symptoms of urinary tract dysfunction and their effect on a patient’s QoL. Only a few of these questionnaires were specially designed for patients with LUTS [6]; out of them, the Neurogenic Bladder Symptom Score (NBSS) [7], the Qualiveen [8], and its short form (SF-Qualiveen) [9] are commonly utilized questionnaires for assessing urinary-specific patients’ QoL with MS.

The NBSS is a questionnaire used to evaluate the symptoms of a neurogenic bladder. It includes 24 questions; the first classifies patients using bladder management methods. The following 22 questions evaluate three domains: voiding, incontinence, and consequences, and the last question addresses the overall QoL [7, 10]. Then, in 2020, Welk et al. developed a short version of NBSS (NBSS-SF) that includes 10 items evaluating the three same domains as the NBSS original version; the NBSS questionnaire is also validated in Arabic [11].

The Qualiveen was initially a French questionnaire [9], translated and validated into English [12], then German [13], Italian [14], Portuguese [15], Spanish [16], and Turkish [17]. The Qualiveen was designed employing expert opinion, interviews with patients, and a literature review. It consists of 30 items in four domains: Bother with limitations, Fears, Feelings, and Frequency of limitations. Later, in 2008, Bonniaud et al. designed a short version of Qualiveen that includes eight questions assessing the four same domains as the Qualiveen version [18]. It is currently available in English [18], German [19], Greek [20] and Polish for MS patients [21]. The aim of our study is to translate, culturally adapt, and validate an Arabic version of the SF-Qualiveen in MS patients to make the SF-Qualiveen suitable for MS patients in both research and clinical practice.

## Methods

### Setting and recruitment of the patients

Adult MS patients with urinary symptomatology who visited the outpatient neurorehabilitation clinics in the Syrian provinces of Damascus and Latakia between September 2022 and March 2023 were invited for the study (our data was collected from four outpatient clinics, two clinics from each province). These facilities specialize in treating people with diseases or nervous system injuries and providing physiotherapy, occupational therapy, and speech and swallowing therapy. Each clinic has a unit for addressing defecation and urination problems caused by neurological injuries. The physical rehabilitation sessions are carried out by qualified therapists under the supervision of physiatrists. All subjects were over 18, had multiple sclerosis, and could read and speak Arabic proficiently. Patients who had undergone urologic surgery within the last twelve months, recently experienced a change in their overall state of health, and experienced a change in their neurogenic bladder (NB) or active urinary infection symptoms within the previous thirty days were excluded. The sample size calculations were determined based on the literature’s recommendation of at least ten patients per question [22]. The SF-Qualiveen had eight questions, so we needed a minimum of eighty MS patients.

The Ethical Committee approved this study in the Al Baath University Institutional Review Board Consent Letter – IRB 2,023,196-S, and all procedures were conducted under the ethical principles outlined in the 1964 Declaration of Helsinki and its subsequent revisions. All our methods were carried out under relevant guidelines and regulations. Informed consent was obtained from all the participants and their legal guardian(s) (illiterate participants). We explained the purpose of the survey to each participant and the way to answer the questionnaire. It was all voluntary; no names were taken, so we provided anonymous data collection.

### Translation and validation of the SF-Qualiveen

This study is divided into two stages. The first one involves translating and cross-culturally adapting the SF-Qualiveen into Arabic. The second one involves assessing the Arabic SF-Qualiveen’s validity and reliability.

### Stage 1: translation and cross-cultural adaptation

The SF-Qualiveen questionnaire has been translated using Beaton et al.‘s self-reported measurement recommendations [23]. The forward translation of the English version of SF-Qualiveen into Arabic was completed by two professional, independent, native Arabic-speaking sworn translators who did not have any clinical knowledge about the project. Then, a first consensus meeting was followed between the two translators and the principal researcher (FK), whose mother language is Arabic. The consensus form was back-translated from Arabic into English by two certified English translators in Arabic who were unaware of the study’s goal or the original versions of the translated questionnaire. The Arabic and English translators, and the principal researcher concurred during the second consensus meeting that the original and the Arabic version were both understandable and equivalent. The final Arabic version was examined by a bilingual panel comprising of two neurorehabilitation specialists (AG, YR), a specialist in the neurologic bladder (AM), and a urogynecologist (SF). The four staff members revised the Arabic version of the questionnaire to evaluate each instrument item’s semantic, idiomatic, and conceptual equivalence. The questionnaire items were reviewed to ascertain their clinical relevance and acceptability for usage in the specific patient group [21]; after completing the assessment, the Arabic version of the SF-Qualiveen was approved by the staff members, and then it was ready for validation.

### Stage 2: validation

#### Reliability

The psychometric properties of the SF-Qualiveen questionnaire were evaluated under the principles of cross-cultural adaptation [23]. Reliability refers to the level of measurement accuracy [24]. This study evaluated both test-retest and internal consistency reliability [24]. Internal consistency is the degree to which items from the same domain correlate. Cronbach’s alpha measures internal consistency, and a score greater than 0.70 can be considered good [25]. For the test-retest of the SF-Qualiveen, all participants completed the SF-Qualiveen at the outpatient clinic (test) and again two weeks later at home (retest). Patients independently completed the NBSS and SF-Qualiveen questionnaires, while the treating physician clarified the precise aim and content of the questionnaires’ items. During this phase of the study, baseline demographic information was also collected. All the patients were asked to complete both questionnaires within 10–15 days to assess test-retest reliability. The interval between both tests was large enough to lower the likelihood that the patients would forget the questions and short enough to avoid any alters in the patients’ bladder management techniques or symptoms. The Intraclass Correlation Coefficient (ICC) was employed to assess the results; an association of more than 0.7 is regarded as strong [25].

#### Validity

Validity indicates if the findings actually reflect what they are trying to measure [24]. In this study, we investigated the construct and content validity. Construct validity refers to an instrument’s capability to evaluate the underlying concept it is looking for [25].This study determined construct validity by evaluating the correlation between the SF-Qualiveen and the NBSS questionnaires using Spearman’s correlation coefficient. Content validity indicates how well an assessment tool covers all relevant items of the construct it aims to measure. It can be assessed by specialists who decide whether the proposed items on the instrument are relevant to the phenomenon to be assessed [25]. Content validity was evaluated through patient interviews; 30 Arabic-speaking MS were asked to evaluate the content validity in face-to-face interviews during September and October 2022, and those patients were not counted from the final sample of our study. All the participants were requested to fill out a questionnaire first. Afterward, the participants were asked to comment on the questions’ content, wording, and language. Furthermore, all the participants were asked if the questions addressed all of their urinary tract problems.

### Statistical analysis

The data are shown as percentages when indicating frequency and categorical data, while the mean and standard deviation were employed for continuous data. Cronbach’s alpha was computed to evaluate the SF-Qualiveen’s internal consistency, with a value of 0.7 or higher being acceptable (from 0.00 to 0.49 = unacceptable; from 0.50 to 0.59 = poor; from 0.60 to 0.69 = questionable; from 0.70 to 0.79 = acceptable; from 0.80 to 0.89 = good; and 0.90 to 1.00 = excellent) [26]. The intraclass correlation coefficient (ICC) was employed to measure test-retest repeatability. The construct validity of the questionnaire was identified by evaluating the association between SF-Qualiveen and NBSS using Spearman’s correlation coefficient. The association’s strength was calculated as follows: (less than 0.10 = negligible; 0.11–0.39 = weak; 0.40–0.69 = moderate;0.70–0.89 = strong; and 0.90–1.00 = very strong correlation [27]. The significance level was fixed at 0.05, and all statistical analyses were computed using SPSS Version 23.0.

## Results

One hundred forty-three patients with symptomatic urinary disorders were asked to participate in this validation study. Thirty-one patients did not respond to the participation invitation. Ten individuals were removed for several reasons: one patient changed treatments during the test-retest interval, nine refused to complete the second questionnaire for unclear reasons. Finally, 102 MS patients completed the second questionnaire (retest), an average of 15.1 ± 8.6 days after the initial set of SF-Qualiveen, and were accepted in the ultimate analyses. The characteristics of the participants with LUTS included in the study are shown in Table 1. Most of them were fully ambulatory 73 (71.6%) with relapsing-remitting MS 64 (62.8%), who voided without catheterization 61 (59.8%) but reported both storage and voiding urinary symptoms 61 (59.8%).

**Table 1:** Demographic and clinical characteristics

Demographic and clinical characteristics	Patients (*n* = 102)
**Age (years)**	44.7 ± 12.4
**Rang of age (years)**	41–65
**Gender**	
Male	31 (30.4%)
Female	71 (69.6%)
**Education levels**	
Illiterate	3 (2.9%)
Elementary School	10 (9.8%)
Middle school	12 (11.8%)
High School	36 (35.3%)
College or more	41 (40.2%)
**MS characteristics**	
**Duration of MS since diagnosis (years)**	14.1 ± 8.4
**MS course**	
Relapsing-remitting	64 (62.8%)
Primary progressive	13 (12.7%)
Secondary progressive	19 (18.6%)
Missing	6 (5.9%)
**Patients Mobility**	
Fully ambulatory	73 (71.6%)
Limited walking without aid	21 (20.6%)
Walking with aid	6 (5.9%)
Wheelchair	2 (1.9%)
**Help needed (Assistance with activities of daily living)**	
At home	19 (18.6%)
Away from home	49 (48.1%)
No need help	34 (33.3%)
**Urinary symptoms**	
**Duration of urinary symptoms (years)**	6.1 ± 5.2
**Type**	
Storage	26 (25.5%)
Voiding	15 (14.7%)
Storage + voiding	61 (59.8%)
**Manner of bladder emptying**	
Normal voiding	61 (59.8%)
Intermittent catheterization	32 (31.4%)
Indwelling catheter	9 (8.8%)

**MS**, multiple sclerosis

### Reliability

#### Internal consistency

The internal consistency for the overall SF-Qualiveen showed a good internal consistency (Cronbach’s alpha of 0.88). The domains “Bother with limitations,” “Fear,” “Feeling,” and “Frequency of limitations” also demonstrated good internal consistency, with a Cronbach’s alpha of > 0.7, as shown in Table 2.

**Table 2:** Internal consistency − Cronbach’s alpha (*n* = 102 MS patients)

	Test	Re-test
**Qualiveen-SF total**	0.90	0.88
**Qualiveen-SF subscales**:		
Bother with limitations	0.83	0.88
Fears	0.71	0.81
Feeling	0.81	0.86
Frequency of limitations	0.68	0.77

Abbreviations: Qualiveen**-SF**, Qualiveen —Short Form

#### Reproducibility

The overall SF- Qualiveen reproducibility was good, with ICCs of 0.91. The ICC value for every SF-Qualiveen domain was higher than 0.7 (0.85 for the Bother with limitations, 0.81 for Fears, 0.86 for Feeling, and 0.81 for Frequency of limitations.), indicating good reproducibility (Table 3).

**Table 3:** Reproducibility of SF-Qualiveen

	Test (mean ± SD)	Re-test (mean ± SD)	Mean change (mean ± SD)	ICC
SF-Qualiveen total score	2.42 ± 0.61	2.45 ± 0.39	0.03 ± 0.41	0.91
Bother with limitations	2.63 ± 0.72	2.64 ± 0.73	0.01 ± 0.52	0.85
Fears	2.31 ± 0.67	2.33 ± 0.84	0.02 ± 0.37	0.81
Feeling	2.83 ± 0.84	2.81 ± 1.02	-0.02 ± 0.37	0.86
Frequency of limitations	2.74 ± 0.84	2.47 ± 0.84	0.00 ± 044	0.81

SD, standard deviation; ICC, intraclass correlation coefficient

### Validity

#### Content validity

Face-to-face interviews with 30 patients with MS were conducted to assess content validity. Most patients agreed that all items were necessary to examine the wide range of bladder problems that patients encounter. The questionnaires were typically accessible, simple to comprehend, and quick to complete for the participating patients, and no changes were required.

#### Construct validity

The construct validity was assessed using Spearman’s rank test, which compared the SF-Qualiveen questionnaire to the NBSS questionnaire. A significant strong association was observed between the QoL item of the NBSS and the SF-Qualiveen overall score (*r* = 0.831, p 0.001) and bother with limitations domain of the SF- Qualiveen (*r* = 0.781, p 0.022). There was a substantial moderate positive association between the overall scores on the NBSS and the domains of the SF- Qualiveen, involving bother with limitations (*r* = 0.473, *p* = 0.027), fears (*r* = 0.611, *p* = 0.031), feelings (*r* = 0.572, *p* = 0.04), and frequency of limitations (*r* = 0.514, *p* = 0.013). The majority of the SF-Qualiveen domain demonstrated a moderate association with the QoL and the storage and voiding domains. The results of SF- Qualiveen showed weak correlation scores for the consequences domains of NBSS (Table 4).

**Table 4:** Correlation between SF-Qualiveen and NBSS scores

		NBSS, total score	NBSS, incontinence domain	NBSS, storage, and voiding domain	NBSS, consequences domain	NBSS, quality of life item
**Qualiveen-SF, total score**	r	0.813**	0.461	0.641**	0.412	0.831**
	P	0.003	0.621	0.002	0.325	0.001
**Qualiveen-SF, bother with limitations domain**	r	0.473*	0.631**	0.591*	0.571	0.781*
	P	0.027	0.001	0.02	0.311	0.022
**Qualiveen-SF, fears domain**	r	0.611*	0.427	0.471*	0.311	0.61*
	P	0.031	0.091	0.01	0.091	0.041
**Qualiveen-SF, feeling domain**	r	0.572*	0.217	0.583*	0.452	0.712*
	P	0.04	0.121	0.013	0.241	0.027
**Qualiveen-SF, frequency of limitations domain**	r	0.514*	0.411	0.315	0.537	0.371
	P	0.013	0.171	0.237	0.091	0.094

Abbreviations: Qualiveen**-SF**, Qualiveen —Short Form; r, spearman’s rho correlation coefficient

∗Significant correlation at *p* < 0.05 (bilateral)

∗∗Significant correlation at *p* < 0.01 (bilateral)

## Discussion

This study validated and culturally adapted the SF-Qualiveen after translating it into Arabic for use among MS patients. The psychometric characteristics presented good statistical results and showed that the Arabic version of the SF-Qualiveen is a consistent, valid, and reliable instrument for use among MS patients with lower urinary tract symptoms. The psychometric properties of the SF-Qualiveen questionnaire enable its use for future research and clinical practice in Arabic countries. The QoL of patients suffering from chronic diseases such as MS is a significant component of healthcare. The symptoms of lower urinary tract problems considerably affect these patients’ quality of life [16]. As a result, employing questionnaires for patients with urinary symptoms is strongly suggested as an essential supplement in LUTS management and shared decision-making with patient-reported outcomes [5, 6]. We decided to validate and translate the SF- Qualiveen since it is practical, simpler to use in clinical practice and research, and less complicated for patients to complete compared to the Qualiveen questionnaire. The length of the questionnaire was mentioned as a limitation in the previous studies, which evaluated the validity of the Qualiveen questionnaire [15, 16].

The SF-Qualiveen was created to facilitate large-scale population surveys by reducing data-gathering time and cost [18]. The validations of the SF-Qualiveen in earlier research tend to be conducted with patient from various centers or patients with various neurological problems [14, 15]. Getting an effective and sufficient number of people with neurogenic disorders to conduct validation is difficult and often impossible. This study tried to validate the SF‐Qualiveen with the involvement of MS patients. The Arabic SF-Qualiveen showed an internal consistency (Cronbach’s alpha = 0.89), similar to the previous validation studies for MS patients (German = 0.85) [19], (Russia = 0.89) [28], and (Polish = 0.86), [21]. The internal consistency of the feelings (0.81) and bother with limitations (0.83) domains was better than the frequency of limitations (0.68) and fears (0.71) domains. A comparable tendency was previously observed by E. S. Philippova et al. for the Russian version (0.79 and 0.93 vs. 0.43 and 0.69, respectively) [28], and Reurs et al. for the German version (0.77 and 0.72 vs. 0.43 and 0.26, respectively) [19], but not mentioned by Przydacz et al. for the Polish SF-Qualiveen (0.81 and 0.89 vs. 0.80 and 0.84, respectively) [21]. Regarding test–retest reliability, we found an ICC of 0.91 similar to those found in the SF-Qualiveen English (0.88) [15], German (0.90) [16], Russia (0.89) [28], and Polish (0.89) [21] version validation studies.

The association between SF-Qualiveen and NBSS scores in patients with MS has never been evaluated before. This association demonstrates the questionnaire’s external validity and its relationship to clinical symptoms. The NBSS is a frequently utilized evaluation tool in neuro-urology; nonetheless, it contains only one item about the QoL [7, 10]. The SF-Qualiveen assesses life satisfaction in terms of lower urinary tract dysfunction. These two instruments complement one another when used in combination.

This study has some limitations, firstly inability to evaluate the responsiveness of the SF-Qualiveen, but other investigations studies have already established this characteristic [14]. Secondly, all participants were recruited in four outpatient clinics from two Syrian provinces, but the participants in rural and distant locations may require a different approach. However, the questionnaire demonstrated good measurement characteristics in the original study and previous validation studies [18, 19, 21].

## Conclusion

The outcomes of this study demonstrate that the SF-Qualiveen is a valid and reliable instrument for evaluating urological dysfunction-related quality of life in the Arabic population with MS. Employing the SF-Qualiveen in their native language can help these patients and their physicians better comprehend bladder and urinary symptoms in regular practice. However, we need additional replication and validation of the results in greater, more varied populations. Future research should, therefore, concentrate on analyzing a large number of participants in the validation study and other psychometric features, like measurement invariance between groups with varied levels of education and health literacy.

## Data Availability

The data collected in this study are available from the corresponding author upon an adequate request.
